# Utility of a simplified ultrasound assessment to assess interstitial pulmonary fibrosis in connective tissue disorders - preliminary results

**DOI:** 10.1186/ar3446

**Published:** 2011-08-18

**Authors:** Marwin Gutierrez, Fausto Salaffi, Marina Carotti, Marika Tardella, Carlos Pineda, Chiara Bertolazzi, Elisabetta Bichisecchi, Emilio Filippucci, Walter Grassi

**Affiliations:** 1 Clinica Reumatologica, Via dei Colli 52, 60035, Università Politecnica delle Marche, Jesi,Ancona, Italy; 2 S.O.D Radiologia Clinica, Dipartimento di Scienze Radiologiche, Via Conca 1, PC 60126 Università Politecnica delle Marche, Ancona, Italy; 3 Instituto Nacional de Rehabilitacion, Av. México-Xochimilco 289, Arenal de Guadalupe, Tlalpan 14389, Mexico City, Mexico

## Abstract

**Introduction:**

Interstitial pulmonary fibrosis (IPF) is a frequent manifestation in patients with connective tissue disorders (CTD). Recently the ultrasound (US) criterion validity for its assessment has been proposed; however, the US scoring systems adopted include the study of several lung intercostal spaces (LIS), which could be time-consuming in daily clinical practice. The aim of this study was to investigate the utility of a simplified US B-lines scoring system compared with both the US comprehensive assessment and the high-resolution computed tomography (HRCT) findings of IPF in CTD patients.

**Methods:**

Thirty-six patients with a diagnosis of CTD were enrolled. Each patient underwent chest HRCT and lung US by an experienced radiologist and rheumatologist, respectively. Both comprehensive and simplified US B-lines assessments were scanned. The comprehensive US assessment was performed at 50 LIS level, whereas the simplified US assessment included bilaterally 14 LIS; for the anterior chest: the second LIS along the para-sternal lines, the fourth LIS along the mid-clavear, anterior axillary and mid-axillary lines; for the posterior chest: the eighth LIS along the paravertebral, sub-scapular and posterior axillary lines.

For criterion validity, HRCT was considered the gold standard. Feasibility, inter and intra-observer reliability was also investigated.

**Results:**

A highly significant correlation between comprehensive and simplified US assessment was found (*P *= 0.0001). A significant correlation was also found between the simplified US assessment and HRCT findings (*P *= 0.0006). Kappa values for the inter-observer simplified US assessment were in a range from 0.769 to 0.885, whereas the concordance correlation coefficient values for the intra-observer were from 0.856 to 0.955. There was a relevant difference in time spent on comprehensive (mean 23.3 ± SD 4.5 minutes) with respect to the simplified US assessment (mean 8.6 ± SD 1.4) (*P *< 0.00001).

**Conclusions:**

Our results provide a new working hypothesis in favor of the utility of a simplified US B-lines assessment as an adjunct method to assess IPF in patients with CTD.

## Introduction

Interstitial pulmonary fibrosis (IPF) is a frequent manifestation in patients with connective tissue disorders (CTD) [1]. The severity of lung involvement may vary considerably depending on the underlying disease and frequently it can be the cause of death of these patients [1,2].

The role of lung ultrasound (US) in the assessment of a variety of pulmonary conditions has been reported previously [3-12]. Only recently has it been proposed as a criterion validity for the assessment of IPF in patients with CTD [13,14] compared with high-resolution computed tomography (HRCT) as the concurrent "gold standard". The US assessment of IPF is determined by the detection and quantification of B-lines, which consist of tails generated by the reflection of the US beam from thickened sub-pleural interlobar septa detectable in between the lung intercostal spaces (LIS) [3,13].

To date, diverse US scoring systems to assess pulmonary diseases, including systemic sclerosis, have been proposed [13-15], but all of these with extensive assessments include the study of several LIS. A comprehensive US B-lines scoring system may be time consuming for both the physician and patient in daily clinical practice. Moreover, its application may impair the follow-up of these patients. On the other hand, at present, there is not enough evidence about which LIS could be better studied for the detection of US B-lines. Therefore, for its feasible overall assessment, the determination of which LIS should be evaluated is necessary. In this way, the development of a novel and valid simplified method for assessing the US B-lines is a challenge absolutely essential in both daily practice and clinical trials. Thus, we decided to investigate the utility of a simplified US B-lines scoring system compared with both the US comprehensive assessment and HRCT findings of IPF in patients with CTD.

## Materials and methods

### Patients

Thirty-six consecutive patients (32 females and 4 males) with a diagnosis of CTD (28 systemic sclerosis, 2 Sjögren's syndrome, 1 undifferentiated CTD, 2 anti-synthetase syndrome, 2 dermatomyositis, 1 mixed CTD), were included in the present study. The diagnoses were made according to the respective international criteria. Mean ± SD age was 57 ± 13 years (range 20 to 78 years) and the mean ± SD disease duration was 88 ± 83.1 months (range 4 to 252 months).

Inclusion criteria were: confirmed diagnosis of CTD and suspicion of IPF, age > 18 years, and chest HRCT performed no longer than 10 days prior to the beginning the study. Patients with a history of pulmonary neoplasia or other causes of interstitial fluid, such as, heart failure, diastolic dysfunction, asthma or pulmonary edema were excluded from the study. All the patients were objects of a multidisciplinary team evaluation composed of cardiologists, pneumologists and rheumatologists. The exclusion of other causes of interstitial fluid was made mainly on the basis of clinical aspects; in those patients with a minimal suspicion of IPF an echocardiogram was made to exclude cardiac involvement.

All patients were attending the out-patient and in-patient clinics of the Rheumatology Department of the Università Politecnica delle Marche (Ancona-Italy).

### Study design

Chest HRCT and US examinations were carried out at the Radiology and Rheumatology Departments of the Università Politecnica delle Marche.

All chest HRCT examinations were performed by two experienced radiologists (EB and MC) and were successively scored by the radiologists, who were experts on HRCT interstitial lung disease (MC) and blinded to the clinical data.

All US examinations were performed by an experienced rheumatologist (MG), who has eight years of experience in musculoskeletal and four years of experience in US lung assessment, and was blinded to both clinical and HRCT findings. Moreover, patients were asked not to talk about their clinical condition with the HRCT and US examiners.

A second rheumatologist sonographer (MT), with two years of experience in musculoskeletal US and one year of lung assessment, was blinded with respect to the first sonographer's and HRCT findings. MT carried out lung US examinations in all patients in order to assess the inter-observer agreement. Prior to the study, the investigators reached a consensus on the adoption of the US scanning technique and the interpretation of US findings. Additionally, a pulmonary function test, which measured single-breath diffusing capacity for carbon monoxide (DLco) was used.

The study was conducted according to the Declaration of Helsinki and local regulations. Ethical approval for the study was obtained from the local Ethics Committee and informed consent was obtained from all patients.

### Chest HRCT assessment

HRCT examination was performed by standard protocol using a CT 64 E light Speed VCT power scanner with a rotation tube with a scanning time of 0.65 seconds. Scans were obtained at full inspiration from the apex to the lung base with the patients in the supine position, at 120 kV and 300 mAs, with a slice thickness of 1.25 mm and slice spacing of 7 mm. The scans were then reconstructed with a HR "bone" algorithm (window level, -500 to -600 HU; window width, 1,800 to 200 HU). In cases showing increased opacification in the postero-basal segments, a limited number of sections were also acquired through the lower zones of the lung, with the patient in the prone position, to ensure that opacification was not due to gravity-dependent perfusion. HRCT assessment does not include the use of contrast media agents.

Pulmonary involvement was evaluated by lung segments according the Warrick score [16]. To correlate accurately the US with HRCT findings it has been expressed in the following semi-quantitative scoring: [0 = normal (0 points); 1 = mild (< 8 points); 2 = moderate (from 8 to 15 points) and 3 = marked (> 15 points).

### US examination

US scanning was performed using a MyLab 70 XVG (Esaote S.p.A., Genoa, Italy) equipped with a 2 to 7 MHz broad band convex multi-frequency transducer.

At the first session, each patient underwent a comprehensive US assessment including anterior, medial and posterior aspects of the chest wall. The anterior chest wall was defined from clavicles to diaphragm and from the sternum to anterior axillary line. The medial chest wall was delineated from armpit to diaphragm and from the anterior to posterior axillary line, whereas the posterior chest wall was defined from a line passing between the 1^st ^and 10^th ^dorsal spinal apofisis, from the posterior axillary line to the paravertebral line.

The comprehensive US assessment was performed in a total of 50 LIS. The assessment of anterior right chest was performed from the second to fifth LIS along the para-sternal, mid-clavear, axillary anterior and mid axillary chest lines, whereas an assessment from the second to fourth LIS along the same lines was performed for the anterior left chest as previously proposed [13-15,17]. An assessment of the left fifth LIS was not performed, since the heart blocks correct visibility of the wall interface.

At the posterior chest level the US examination was obtained from the second to eighth LIS along the paravertebral lines and from the seventh to eighth LIS along both the sub-scapular and axillary posterior lines. Each LIS was scanned in a longitudinal scan moving the probe from the medial to lateral part along the anatomical references lines to enable maximum coverage of the anatomical surface area. US Greyscale imaging parameters were set in order to obtain the maximal contrast among all the structures under examination.

Patient positions were supine or near-supine for the anterior chest scanning, while in a sitting position for the posterior chest scanning.

The second time, we obtained a simplified US B-lines model which consisted of a total of 14 LIS bilateral scans. For the anterior chest, the authors considered the second LIS along the para-sternal lines, the fourth LIS along the mid-clavear, the anterior axillary and the mild-axillary lines. For the posterior chest, the eighth LIS along the paravertebral, the sub-scapular and the posterior axillary lines were selected. The simplified score was obtained by a simple *post-hoc *analysis resulting from US comprehensive assessment. The 14 sites were chosen because they demonstrated both higher prevalence of US B-lines in the comprehensive assessment and easy accessibly by US.

The respective sites assessed by US for both comprehensive and simplified assessment are represented in Table 1. Moreover, the time spent with each patient for both US B-lines systems was recorded.

**Table 1:** Anatomical sites assessed by comprehensive and simplified US B-lines assessment

	Anatomical lines	Comprehensive US B-lines assessment		Simplified US B-lines assessment	
		**Right**	**Left**	**Right**	**Left**

	**para-sternal**	2^nd^, 3^rd^, 4^th^, 5^th ^LIS	2^nd^, 3^rd^, 4^th ^LIS	2^nd ^LIS	2^nd ^LIS
**ANTERIOR**	**mid-clavear**	2^nd^, 3^rd^, 4^th^, 5^th ^LIS	2^nd^, 3^rd^, 4^th ^LIS	4^th ^LIS	4^th ^LIS
	**anterior axillary**	2^nd^, 3^rd^, 4^th^, 5^th ^LIS	2^nd^, 3^rd^, 4^th ^LIS	4^th ^LIS	4^th ^LIS
	**mid-axillary**	2^nd^, 3^rd^, 4^th^, 5^th ^LIS	2^nd^, 3^rd^, 4^th ^LIS	4^th ^LIS	4^th ^LIS
	**paravertebral**	2^nd^, 3^rd^, 4^th^, 5^th,^, 6^th^, 7^th^,8^th ^LIS	2^nd^, 3^rd^, 4^th^, 5^th,^, 6^th^, 7^th^, 8^th ^LIS	8^th ^LIS	8^th ^LIS
**POSTERIOR**	**sub-scapular**	7^th^, 8^th ^LIS	7^th^, 8^th ^LIS	8^th ^LIS	8^th ^LIS
	**Posterior axillary**	7^th^, 8^th ^LIS	7^th^, 8^th ^LIS	8^th ^LIS	8^th ^LIS

LIS, lung intercostal spaces; US, ultrasound.

### US interpretation

**Figure 1:**
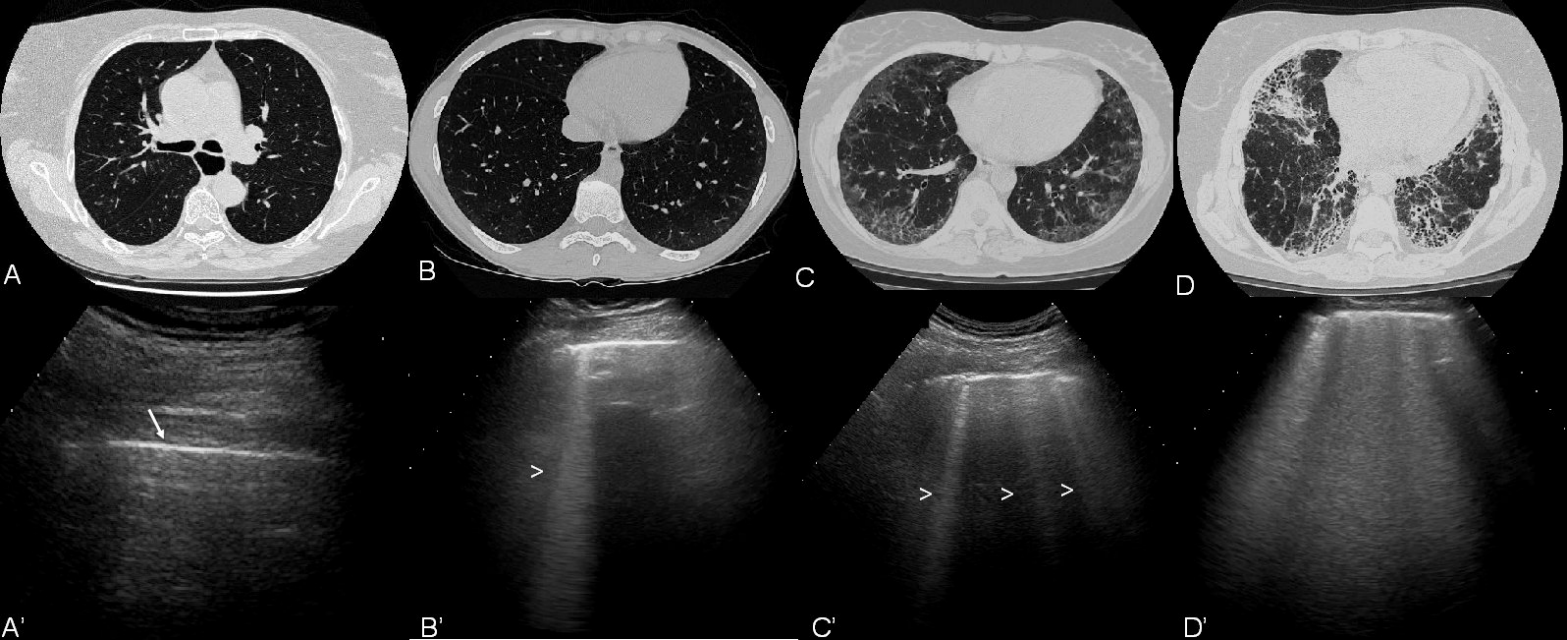
**HRCT features of interstitial pulmonary fibrosis**. **A**. Normal aspect of the lung. **B**. Mild. **C**. Moderate. **D**. Severe. **A'**. US examination of healthy interlobular septa at lung surface level. Note as the pleura is a linear and regular hyperechoic band (arrow). **B'-D'**. US examinations showing different scores of fibrotic pulmonary involvement: **B'**. Mild. **C'**. Moderate. **D'**. Severe.

The elementary finding evaluated was the US B-line, an artifact generated from the thickened interlobular septa at lung surface level. It was defined as a hyperechoic narrow-based reverberation type of artifact, spreading like a laser-ray up to the edge of the screen. The US B-lines generally are not present in healthy lungs [7,18] (Figure 1A).

In each LIS, the number of US B-lines was recorded. Subsequently, the US B-lines total sum of all LIS was recorded and graded according a semi-quantitative scoring to correlate with HRCT findings (Figure 1). For the comprehensive assessment the semi-quantitative score was 0 = normal, (< 10 B-lines); 1 = mild (from 11 to 20 B-lines); 2 = moderate (from 21 to 50 B-lines) and 3 = marked (> 50 B-lines) whereas for the *simplified assessment *the semi-quantitative score was 0 = normal, (< 5 B-lines); 1 = mild (from 6 to 15 B-lines); 2 = moderate (from 16 to 30 B-lines) and 3 = marked (> 30 B-lines). The semi-quantitative scoring was obtained employing the distribution of percentiles analysis.

### US B-lines intra-observer reliability

The B-lines intra-observer reliability was assessed by recording representative dynamic clips of the full simplified baseline examination of all patients involved in the study. The stored images of each patient were blindly scored by the same investigator (MG) who successively performed the corresponding US lung examination two weeks after the baseline assessment.

### Statistical analysis

Statistical analysis was performed using MedCalc, version 10.0 (MedCalc Software, Mariakerke, Belgium). Standard descriptive results were expressed as mean and standard deviation (SD) whereas the categorical data were expressed as proportions. Chi square analysis was used for the comparison between the US and HRCT data, whereas the Spearman's rho correlation coefficient was used for the respective correlation. *P*-values below 0.05 were considered statistically significant. A scatter plot graph was used to demonstrate the correlation between comprehensive and simplified US assessments.

To assess intra-observer and inter-observer reliability between the two investigators, the terms of semi-quantitative scoring were calculated by a weighted kappa statistic. A kappa value of 0 to 0.20 was considered poor, 0.21 to 0.40 fair, 0.41 to 0.60 moderate, 0.61 to 0.80 good and 0.81 to 1.00 excellent [19].

The feasibility of simplified US B-lines was estimated by comparing the time spent with respect to comprehensive assessment by the independent samples *t*-test. A *P-*value less than 0.005 was considered statistically significant.

## Results

A total of 1,700 LIS was assessed for the comprehensive US B-lines assessment, whereas 476 LIS were evaluated for the simplified US assessment in 36 patients. Twenty-one (58.3%) patients showed a grade 3 of IFP according to the Warrick score. Five (13.8%) patients showed a grade 2, and 2 (5.5%) a grade 1. Eight patients did not show HRCT signs of IPF. In these patients the HRCT was performed on the basis of the results of a pulmonary function test, which showed alterations in the single-breath diffusing capacity for carbon monoxide (DLco).

A positive correlation was found between the US B-lines assessment and Warrick score HRCT assessment in both comprehensive and simplified methods (*P *= 0.0006). Moreover a higher significant correlation between the comprehensive (50 sites) and simplified scoring systems (14 sites) was detected (Spearman's rank test *P *= 0.0001).

The global kappa values for the inter-observer reliability of comprehensive US semi-quantitative assessment at para-sternal, mid-clavear, anterior axillary, mid-axillary, paravertebral, sub-scapular and posterior axillary level were: 0.943, 0.846, 0.963, 0.932, 0.958, 0.969 and 0.980 respectively.

Moreover, kappa values of simplified US semi-quantitative assessments of inter-observer showed a good agreement between the two investigators (Table 2). The kappa values for the intra-observer reliability of simplified US B-lines assessment are reported in Table 2.

**Table 2:** Inter-and intra-observer agreement data for simplified US B-lines assessment

	Inter-observer	Intra-observer
Anatomical lines	weighted kappa values for the semiquantitative system	weighted kappa values for the semiquantitative system
**2^nd ^para-sternal LIS**	0.885	0.864
**4^th ^mid-clavear LIS**	0.836	0.881
**4^th ^anterior axillary LIS**	0.863	0.868
**4^th ^mid-axillary LIS**	0.812	0.845
**8^th ^paravertebral LIS**	0.769	0.894
**8^th ^sub-scapular LIS**	0.828	0.883
**8^th ^posterior axilary LIS**	0.864	0.862

Agreement between both sonographers on both the comprehensive simplified US assessment.

A significant difference between the mean time spent on the comprehensive US B-lines assessment (mean 23.3 ± SD 4.5, range 16 to 31 minutes) and the mean time spent on the simplified US B-lines assessment (mean 8.6 ± SD 1.4, range 6 to 12 minutes, *P *< 0.00001) was found.

## Discussion

To detect and quantify IPF in patients with CTD represents one of the primary goals in order to improve the quality of life of these patients [1,20-24].

Currently, chest HRCT is considered the "gold-standard" for the diagnosis, disease activity and therapy monitoring of IPF. Its value is remarkable since it has been demonstrated also to be able to detect both early pulmonary changes and subclinical lung involvement [21,24].

Although chest US is used to assess different lung conditions, such as pulmonary interstitial edema or congestion, heart and respiratory failure, atelectasis, pleural effusions, and to guide interventional chest procedures, such as thoracentesis or pleural lesion biopsy [2-12], its potential role in the assessment of IPF has been recently proposed in patients with systemic sclerosis [13,14]. The results of these studies are encouraging since they demonstrate a good correlation with HRCT as a concurrent "gold standard". This opens up an interesting window of research focused on the US B-lines as surrogate biomarkers of pulmonary changes in patients with CTD.

US offers particular characteristics for the chest assessment. From the practical viewpoint: it is a bedside procedure widely available, inexpensive, readily and largely accepted by the patient. From the technical viewpoint: first, the surface of the lung can be easily studied by US, so the B-lines "artifacts" are quickly detected; second, although small surface probes with frequencies range between 3 to 3.5 MHz were quite suitable for this specific assessment, transducers with large surface and frequencies between 5 to 7.5 MHz can be equally valuable, as recently demonstrated by Delle Sedie *et al*. [25]. Finally, portable machines even without Doppler power can be sufficient for a complete and detailed lung assessment.

In spite of these innovative data, the current US scoring systems proposed to assess the B-lines are extensive, including the study of 50 or more LIS, which is time-consuming in daily clinical practice and difficult to make the comparison of multi-center study results [13-15,17]. Additionally, they have not taken into account a semi-quantitative assessment which can facilitate the interpretation of the collected data. Thus, we decided to test a simplified US semi-quantitative scoring for assessing the B-lines in patients with CTD.

The simplified US B-lines assessment is composed of 14 LIS, chosen on the basis of the major prevalence of US B-lines detected during the comprehensive assessment, their easy accessibility and their covering of main pulmonary segments involved with IPF.

Our results showed a significant correlation with both comprehensive US B-lines assessment and HRCT findings. To the best of our knowledge this is the first study providing evidence in favor of the utility of this novel, simplified US B-lines assessment. Results of inter and intra-observer reliability were also highly significant.

The mean time spent in performing a simplified US B-lines examination for each patient was much less in respect to comprehensive assessment (8.3 minutes versus 23.3 minutes respectively). To our knowledge this remains a controversial point since some authors previously indicated that a comprehensive US B-lines assessment could be performed in less than 10 minutes [13,14]. Probably this is true for patients with mild IPF, which is characterized by little quota of US B-lines. In fact, most patients included in these studies did not have a high Warrick score. In our study, we included 19 patients (55.8%) with severe IPF characterized by a high Warrick score. Although the count of B-lines was more difficult in this group, since it required both more attention and more time, we believe that the inclusion of patients with whole ranges of degrees of IPF may give more accurate information about the reproducibility as well as feasibility for patient follow-up. Our study takes into account patients with different CTD. In order to avoid facts that can negatively influence the study, the sonographic features should be interpreted in the light that they not provide results in a disease driven manner but in an anatomic driven way.

The main limitation of our study is the low number of enrolled patients, which does not permit an accurate evaluation in terms of sensitivity and specificity which could more strongly support these data.

HRCT remains the gold-standard used to assess IPF, since it is the only imaging method that gives information about the whole lung, and is not limited to the subpleural interstitial lobular septa. Despite this, we believe that US can be used as an adjunct method in the assessment of monitoring of lung disease evolution. Additional advantages of US consist of its low cost, the fact that it can also be performed at the bedside and that it is a non-ionizing technique. This last aspect is fundamental, especially in patients who need serial examinations for monitoring disease progression. Besides, it can play a relevant role for screening purposes aimed towards the early identification of patients that require a chest HRCT. Nevertheless, additional investigations studying a larger series of cohorts, including sensitivity and specificity and a stratification of Warrick score into fibrosis and alveolitis to demonstrate which correlates better with HRCT findings, may be useful to more strongly support these observations. In particular, a focus aimed at determining sensitivity to change during the progression of IPF could provide precious information about the responsiveness of the simplified US assessment.

## Conclusions

The results of the present study provide a new working hypothesis that a simplified US B-lines assessment may be an additional, useful imaging method in the evaluation of IPF in CTD patients.

## Abbreviations

CTD: connective tissue disorders; DLco: diffusing capacity for carbon monoxide; HRCT: high-resolution computed tomography; IPF: interstitial pulmonary fibrosis; LIS: lung intercostal spaces; SD: standard deviation; US: ultrasound

## Competing interests

The authors declare that they have no competing interests.

## Authors' contributions

MG participated in the study development, recruitment of patients, performed the ultrasound examinations (sonographer 1), prepared the sonographic images, conducted data evaluation and prepared the manuscript. FS participated in the statistical analysis and data evaluation and manuscript preparation. MC performed the HRCT exams, prepared the HRCT images, conducted data evaluation and prepared the manuscript. MT performed the ultrasound examinations (sonographer 2) and gave substantial input to data evaluation and manuscript preparation. CP gave substantial input to the data evaluation and manuscript preparation. CB participated actively in the recruitment of patients and manuscript preparation. EF participated in the study development and gave substantial input to the data evaluation and manuscript preparation. WG participated in the study development and gave substantial input to the data evaluation and manuscript preparation. All authors read and approved the final version of manuscript.

## Acknowledgements

Written consent to publish was obtained from the patients.
